# Crystal structure of (*Z*)-3-benz­yloxy-6-[(2-hy­droxy­anilino)methyl­idene]cyclo­hexa-2,4-dien-1-one

**DOI:** 10.1107/S1600536814024568

**Published:** 2014-11-26

**Authors:** Nadir Ghichi, Ali Benboudiaf, Hocine Merazig

**Affiliations:** a Unité de Recherche de Chimie de l’Environnement et Moléculaire Structurale (CHEMS), Faculté des Sciences Exactes, Département de Chimie, Université Constantine 1, Algeria

**Keywords:** crystal structure, pharmaceutical applications, industrial applications, azomethines, hydro­philicity, drug properties, hydrogen bonding, C—H⋯π inter­actions

## Abstract

In the title compound, C_20_H_17_NO_3_, the methyl­idene­cyclo­hexa-2,4-dienone moiety is approximately planar [maximum deviation = 0.0615 (10) Å] and is oriented at diherdral angles of 69.60 (7) and 1.69 (9)° to the phenyl and hy­droxy­benzene rings, respectively. The amino group links with the carbonyl O atom *via* an intra­molecular N—H⋯O hydrogen bond, forming an *S*(6) ring motif. In the crystal, the mol­ecules are linked by O—H⋯O hydrogen bonds and weak C—H⋯O and C—H⋯π inter­actions, forming a three-dimensional supra­molecular architecture.

## Related literature   

For pharmaceutical and industrial applications of azomethines, see: Prakash & Adhikari (2011 ▶). For the effect of hydro­philicity on drug properties, see: Lin & Lu (1997 ▶).
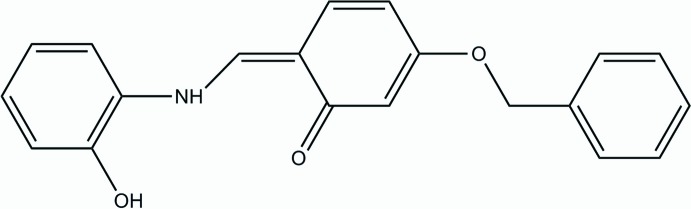


## Experimental   

### Crystal data  

C_20_H_17_NO_3_
*M* *_r_* = 319.21
Monoclinic, 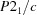

*a* = 12.890 (5) Å
*b* = 8.343 (5) Å
*c* = 19.908 (5) Å
β = 129.616 (15)°
*V* = 1649.2 (12) Å^3^
*Z* = 4
Mo *K*α radiation
μ = 0.09 mm^−1^
*T* = 293 K
0.03 × 0.02 × 0.01 mm

### Data collection  

Bruker APEXII CCD diffractometer
10996 measured reflections
2845 independent reflections
2019 reflections with *I* > 2σ(*I*)
*R*_int_ = 0.026

### Refinement  

*R*[*F* ^2^ > 2σ(*F* ^2^)] = 0.040
*wR*(*F* ^2^) = 0.105
*S* = 1.01
2845 reflections
217 parameters
H-atom parameters constrained
Δρ_max_ = 0.13 e Å^−3^
Δρ_min_ = −0.18 e Å^−3^

### 

Data collection: *APEX2* (Bruker, 2006 ▶); cell refinement: *SAINT* (Bruker, 2006 ▶); data reduction: *SAINT*; program(s) used to solve structure: *SHELXS97* (Sheldrick, 2008 ▶); program(s) used to refine structure: *SHELXL97* (Sheldrick, 2008 ▶); molecular graphics: *ORTEP-3 for Windows* (Farrugia, 2012 ▶); software used to prepare material for publication: *WinGX* (Farrugia, 2012 ▶).

## Supplementary Material

Crystal structure: contains datablock(s) global, I. DOI: 10.1107/S1600536814024568/xu5827sup1.cif

Structure factors: contains datablock(s) I. DOI: 10.1107/S1600536814024568/xu5827Isup2.hkl

Supporting information file. DOI: 10.1107/S1600536814024568/xu5827Isup3.cml

**Fig. 1.**
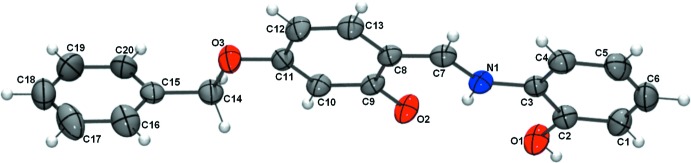
View of the molecular structure of the title compound, with atom labelling. Displacement ellipsoids are drawn at the 50% probability level.

**Fig. 2.**
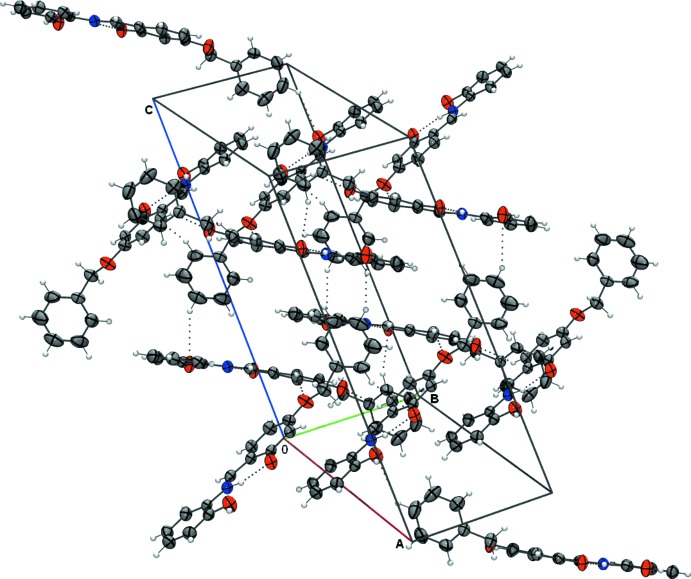
Partial view along the b axis of the crystal packing of the title compound, showing the hydrogen bonds as dashed lines (see Table 1 for details).

. DOI: 10.1107/S1600536814024568/xu5827fig1.tif
View of the mol­ecular structure of the title compound, with atom labelling. Displacement ellipsoids are drawn at the 50% probability level.

b . DOI: 10.1107/S1600536814024568/xu5827fig2.tif
Partial view along the *b* axis of the crystal packing of the title compound, showing the hydrogen bonds as dashed lines (see Table 1 for details).

CCDC reference: 1033206

Additional supporting information:  crystallographic information; 3D view; checkCIF report

## Figures and Tables

**Table 1:** Hydrogen-bond geometry (, ) *Cg*1 is the centroid of the C15C20 ring.

*D*H*A*	*D*H	H*A*	*D* *A*	*D*H*A*
N1H01O2	0.94	1.81	2.594(2)	139
O1H3O2^i^	0.82	1.75	2.563(3)	170
C13H13O3^ii^	0.93	2.53	3.309(3)	141
C14H14*A* *Cg*1^iii^	0.97	2.66	3.406(3)	134

Symmetry codes: (i) 
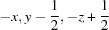
; (ii) 
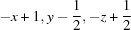
; (iii) 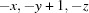.

## supplementary crystallographic information

### S1. Comment

Azomethine compounds are extensively incoporated in many pharmaceutical and food industry applications (Prakash & Adhikari, 2011). Elimination of excess drugs from the bloodstream or body is an essential process to protect against potential toxicity. In most cases the more hydrophilic drugs/pharmacophores are the more they are readily excreted by the kidneys in urine (Lin & Lu, 1997). The existence of conjugated double bonds and more hydroxylic groups in bioactive molecules increases not only their hydrophilicity but also the rate of their membrane absorption. Based on such facts we herein report the crystal structure of a potential bioactive hydrophilic azomethine derivative.

A view of the molecular structure of (I) with numbering Scheme is shown in Fig. 1. In the molecule, the methylene-cyclohexa-2,4-dienone moiety is approximately planar [maximum deviation = 0.0615 (10) Å] and its mean plane is oriented with respect to the terminal benzene rings at 69.60 (7) and 1.69 (9)°, respectively. The amino group links with the carbonyl O atom via an intramolecular N—H···O hydrogen bond, forming an S(6) ring motif. In the crystal, the molecules are linked by the intermolecular O—H···O hydrogen bond, weak C—H···O and C—H···π interactions to form the three dimensional supramolecular architecture.

### S2. Experimental

A mixture of 2-aminophenol (1 mmol), and 4-(benzyloxy)-2-hydroxybenzaldehyde (1 mmol) was added and heated to form a clear solution. To this a few drops of conc. HCL was added as a catalyst and refluxed for 4 h. After cooling the solution, After stirring at 80°C for 45 min the formed precipitate was filtered off and washed with ice ether and ethyl acetate to give pure Schiff base as an Orange solid in an 35% yield. The crude product was dissolved in ethyl acetate and two spoons of activated charcoal were added. the mixture was filtered and the product was crystallized from an ethyl acetate solution.

### S3. Refinement

All hydrogen atoms were fixed geometrically and treated as riding with C—H = 0.93–0.97 Å and N—H = 0.86 Å, U_iso_(H) = 1.2U_eq_(C,N).

### Crystal data

**Table:**

C_20_H_17_NO_3_	*Z* = 4
*M**_r_* = 319.21	*F*(000) = 672
Monoclinic, *P*2_1_/*c*	*D*_x_ = 1.286 Mg m^−^^3^
Hall symbol: -P 2ybc	Mo *K*α radiation, λ = 0.71073 Å
*a* = 12.890 (5) Å	µ = 0.09 mm^−^^1^
*b* = 8.343 (5) Å	*T* = 293 K
*c* = 19.908 (5) Å	Block, orange
β = 129.616 (15)°	0.03 × 0.02 × 0.01 mm
*V* = 1649.2 (12) Å^3^	

### Data collection

**Table:**

Bruker APEXII CCD diffractometer	2019 reflections with *I* > 2σ(*I*)
Radiation source: fine-focus sealed tube	*R*_int_ = 0.026
Graphite monochromator	θ_max_ = 25.1°, θ_min_ = 2.7°
phi and ω scans	*h* = −15→14
10996 measured reflections	*k* = −9→9
2845 independent reflections	*l* = −23→22

### Refinement

**Table:**

Refinement on *F*^2^	Primary atom site location: structure-invariant direct methods
Least-squares matrix: full	Secondary atom site location: difference Fourier map
*R*[*F*^2^ > 2σ(*F*^2^)] = 0.040	Hydrogen site location: inferred from neighbouring sites
*wR*(*F*^2^) = 0.105	H-atom parameters constrained
*S* = 1.01	*w* = 1/[σ^2^(*F*_o_^2^) + (0.0535*P*)^2^ + 0.1282*P*] where *P* = (*F*_o_^2^ + 2*F*_c_^2^)/3
2845 reflections	(Δ/σ)_max_ < 0.001
217 parameters	Δρ_max_ = 0.13 e Å^−^^3^
0 restraints	Δρ_min_ = −0.18 e Å^−^^3^

### Special details

**Table:**

Geometry. Bond distances, angles *etc*. have been calculated using the rounded fractional coordinates. All su's are estimated from the variances of the (full) variance-covariance matrix. The cell e.s.d.'s are taken into account in the estimation of distances, angles and torsion angles
Refinement. Refinement of *F*^2^ against ALL reflections. The weighted *R*-factor *wR* and goodness of fit *S* are based on *F*^2^, conventional *R*-factors *R* are based on *F*, with *F* set to zero for negative *F*^2^. The threshold expression of *F*^2^ > σ(*F*^2^) is used only for calculating *R*-factors(gt) *etc*. and is not relevant to the choice of reflections for refinement. *R*-factors based on *F*^2^ are statistically about twice as large as those based on *F*, and *R*- factors based on ALL data will be even larger.

### Fractional atomic coordinates and isotropic or equivalent isotropic displacement parameters (Å^2^)

**Table:**

	*x*	*y*	*z*	*U*_iso_*/*U*_eq_	
O1	0.08170 (10)	−0.46817 (14)	0.29489 (8)	0.0510 (4)	
O2	0.13038 (10)	−0.09947 (13)	0.24415 (7)	0.0445 (4)	
O3	0.29081 (10)	0.34273 (14)	0.18059 (7)	0.0497 (4)	
N1	0.29329 (12)	−0.33924 (15)	0.32821 (8)	0.0349 (4)	
C1	0.20033 (17)	−0.7115 (2)	0.37366 (11)	0.0465 (6)	
C2	0.19333 (15)	−0.56093 (19)	0.34232 (10)	0.0368 (5)	
C3	0.30842 (14)	−0.49543 (19)	0.36000 (9)	0.0327 (5)	
C4	0.42609 (15)	−0.5832 (2)	0.40563 (10)	0.0418 (6)	
C5	0.43154 (17)	−0.7336 (2)	0.43620 (11)	0.0469 (6)	
C6	0.31962 (18)	−0.7966 (2)	0.42096 (12)	0.0499 (7)	
C7	0.38268 (14)	−0.2492 (2)	0.33441 (10)	0.0359 (5)	
C8	0.35558 (14)	−0.09631 (19)	0.29854 (10)	0.0334 (5)	
C9	0.22447 (14)	−0.02458 (18)	0.25091 (10)	0.0333 (5)	
C10	0.20234 (14)	0.12504 (19)	0.21107 (10)	0.0364 (5)	
C11	0.30365 (15)	0.2003 (2)	0.21852 (10)	0.0373 (5)	
C12	0.43407 (15)	0.1325 (2)	0.26765 (11)	0.0429 (6)	
C13	0.45764 (14)	−0.0112 (2)	0.30572 (10)	0.0391 (6)	
C14	0.15921 (15)	0.4129 (2)	0.12213 (11)	0.0459 (6)	
C15	0.16707 (14)	0.5500 (2)	0.07665 (10)	0.0413 (6)	
C16	0.1628 (2)	0.7053 (2)	0.09686 (12)	0.0615 (8)	
C17	0.1699 (2)	0.8323 (3)	0.05546 (15)	0.0794 (9)	
C18	0.1801 (2)	0.8034 (3)	−0.00759 (15)	0.0718 (9)	
C19	0.18242 (19)	0.6501 (3)	−0.02999 (14)	0.0675 (9)	
C20	0.17652 (16)	0.5232 (2)	0.01228 (12)	0.0550 (7)	
H1	0.12478	−0.75575	0.36294	0.0558*	
H01	0.20956	−0.28801	0.29816	0.0419*	
H3	0.01953	−0.51531	0.28781	0.0765*	
H4	0.50165	−0.54071	0.41573	0.0501*	
H5	0.51090	−0.79264	0.46715	0.0562*	
H6	0.32429	−0.89733	0.44272	0.0599*	
H7	0.46914	−0.28998	0.36450	0.0430*	
H10	0.11811	0.17324	0.17938	0.0437*	
H12	0.50254	0.18660	0.27351	0.0515*	
H13	0.54313	−0.05599	0.33761	0.0469*	
H14A	0.09445	0.33409	0.07991	0.0551*	
H14B	0.13102	0.45110	0.15431	0.0551*	
H16	0.15485	0.72580	0.13934	0.0738*	
H17	0.16786	0.93702	0.07052	0.0953*	
H18	0.18538	0.88871	−0.03536	0.0861*	
H19	0.18798	0.63063	−0.07361	0.0811*	
H20	0.17896	0.41865	−0.00285	0.0660*	

### Atomic displacement parameters (Å^2^)

**Table:**

	*U*^11^	*U*^22^	*U*^33^	*U*^12^	*U*^13^	*U*^23^
O1	0.0330 (6)	0.0491 (8)	0.0727 (8)	0.0009 (5)	0.0345 (6)	0.0098 (7)
O2	0.0309 (6)	0.0414 (7)	0.0679 (8)	0.0004 (5)	0.0346 (6)	0.0052 (6)
O3	0.0290 (6)	0.0565 (8)	0.0542 (8)	0.0006 (5)	0.0221 (6)	0.0197 (6)
N1	0.0274 (7)	0.0368 (8)	0.0406 (8)	0.0005 (6)	0.0217 (6)	0.0000 (6)
C1	0.0482 (10)	0.0438 (11)	0.0555 (11)	−0.0055 (9)	0.0368 (9)	−0.0001 (9)
C2	0.0342 (9)	0.0397 (10)	0.0399 (9)	0.0004 (8)	0.0252 (8)	−0.0017 (8)
C3	0.0319 (8)	0.0343 (10)	0.0322 (9)	−0.0013 (7)	0.0206 (7)	−0.0037 (7)
C4	0.0333 (9)	0.0445 (11)	0.0432 (10)	0.0003 (8)	0.0224 (8)	−0.0027 (9)
C5	0.0432 (10)	0.0440 (11)	0.0450 (10)	0.0104 (8)	0.0242 (9)	0.0048 (9)
C6	0.0596 (12)	0.0405 (11)	0.0516 (11)	0.0026 (9)	0.0364 (10)	0.0060 (9)
C7	0.0278 (8)	0.0434 (11)	0.0375 (9)	0.0000 (7)	0.0213 (7)	−0.0039 (8)
C8	0.0273 (8)	0.0374 (10)	0.0363 (9)	−0.0027 (7)	0.0207 (7)	−0.0038 (8)
C9	0.0276 (8)	0.0360 (10)	0.0404 (9)	−0.0048 (7)	0.0236 (7)	−0.0071 (8)
C10	0.0248 (8)	0.0411 (10)	0.0409 (10)	0.0020 (7)	0.0198 (7)	0.0014 (8)
C11	0.0303 (8)	0.0431 (10)	0.0368 (9)	−0.0040 (7)	0.0206 (7)	0.0023 (8)
C12	0.0282 (9)	0.0507 (12)	0.0500 (11)	−0.0048 (8)	0.0250 (8)	0.0036 (9)
C13	0.0246 (8)	0.0464 (11)	0.0449 (10)	0.0011 (7)	0.0215 (7)	0.0017 (9)
C14	0.0306 (9)	0.0520 (11)	0.0459 (10)	0.0006 (8)	0.0201 (8)	0.0089 (9)
C15	0.0266 (8)	0.0496 (12)	0.0345 (9)	−0.0029 (8)	0.0134 (7)	0.0049 (8)
C16	0.0778 (14)	0.0555 (14)	0.0382 (11)	−0.0166 (11)	0.0309 (10)	−0.0072 (10)
C17	0.1057 (18)	0.0477 (14)	0.0607 (15)	−0.0198 (12)	0.0418 (14)	−0.0049 (12)
C18	0.0623 (13)	0.0730 (17)	0.0628 (15)	−0.0103 (12)	0.0318 (12)	0.0223 (13)
C19	0.0553 (12)	0.0960 (19)	0.0647 (14)	0.0165 (12)	0.0445 (11)	0.0287 (14)
C20	0.0482 (11)	0.0627 (13)	0.0620 (12)	0.0134 (9)	0.0388 (10)	0.0119 (11)

### Geometric parameters (Å, º)

**Table:**

O1—C2	1.352 (2)	C15—C20	1.381 (3)
O2—C9	1.293 (3)	C15—C16	1.368 (3)
O3—C11	1.360 (2)	C16—C17	1.381 (3)
O3—C14	1.434 (3)	C17—C18	1.364 (4)
O1—H3	0.8200	C18—C19	1.361 (4)
N1—C7	1.314 (3)	C19—C20	1.384 (3)
N1—C3	1.407 (2)	C1—H1	0.9300
N1—H01	0.9400	C4—H4	0.9300
C1—C6	1.382 (3)	C5—H5	0.9300
C1—C2	1.380 (3)	C6—H6	0.9300
C2—C3	1.399 (3)	C7—H7	0.9300
C3—C4	1.380 (3)	C10—H10	0.9300
C4—C5	1.377 (3)	C12—H12	0.9300
C5—C6	1.375 (4)	C13—H13	0.9300
C7—C8	1.393 (2)	C14—H14A	0.9700
C8—C13	1.420 (3)	C14—H14B	0.9700
C8—C9	1.439 (3)	C16—H16	0.9300
C9—C10	1.407 (2)	C17—H17	0.9300
C10—C11	1.369 (3)	C18—H18	0.9300
C11—C12	1.416 (3)	C19—H19	0.9300
C12—C13	1.346 (2)	C20—H20	0.9300
C14—C15	1.502 (3)		
			
O1···N1	2.594 (3)	C10···H14A	2.6800
O1···O2^i^	2.563 (3)	C14···H10	2.5200
O2···C2^ii^	3.364 (3)	C15···H13^iii^	3.0900
O2···N1	2.594 (2)	C15···H14A^ix^	2.9300
O2···O1^ii^	2.563 (3)	C16···H14A^ix^	2.9300
O3···C13^iii^	3.309 (3)	C17···H14A^ix^	3.0100
O1···H18^iv^	2.8100	C18···H14A^ix^	3.0700
O1···H01	2.2000	C19···H6^x^	3.0600
O2···H16^v^	2.7200	C19···H14A^ix^	3.0400
O2···H1^ii^	2.8500	C20···H13^iii^	2.9400
O2···H3^ii^	1.7500	C20···H14A^ix^	2.9700
O2···H01	1.8100	H1···H3	2.3500
O3···H13^iii^	2.5300	H1···O2^i^	2.8500
N1···O1	2.594 (3)	H01···O1	2.2000
N1···O2	2.594 (2)	H01···O2	1.8100
C1···C10^v^	3.528 (3)	H01···C9	2.4500
C2···O2^i^	3.364 (3)	H3···H1	2.3500
C4···C4^vi^	3.248 (3)	H3···O2^i^	1.7500
C4···C11^v^	3.483 (3)	H3···C9^i^	2.7200
C4···C5^vi^	3.592 (3)	H4···C7	2.7800
C5···C11^v^	3.563 (3)	H4···H7	2.2500
C5···C4^vi^	3.592 (3)	H6···C19^xi^	3.0600
C5···C7^vi^	3.559 (3)	H6···H19^xi^	2.5000
C5···C12^v^	3.556 (4)	H7···C4	2.7500
C6···C10^v^	3.481 (3)	H7···H4	2.2500
C6···C9^v^	3.360 (3)	H7···H13	2.3800
C7···C5^vi^	3.559 (3)	H10···C14	2.5200
C8···C16^v^	3.509 (3)	H10···H14A	2.2500
C9···C16^v^	3.465 (3)	H10···H14B	2.4000
C9···C6^vii^	3.360 (3)	H13···H7	2.3800
C10···C1^vii^	3.528 (3)	H13···O3^viii^	2.5300
C10···C6^vii^	3.481 (3)	H13···C15^viii^	3.0900
C11···C4^vii^	3.483 (3)	H13···C20^viii^	2.9400
C11···C5^vii^	3.563 (3)	H14A···C10	2.6800
C12···C5^vii^	3.556 (4)	H14A···H10	2.2500
C13···O3^viii^	3.309 (3)	H14A···H20	2.5900
C14···C20^ix^	3.377 (4)	H14A···C15^ix^	2.9300
C14···C19^ix^	3.568 (4)	H14A···C16^ix^	2.9300
C14···C15^ix^	3.496 (3)	H14A···C17^ix^	3.0100
C15···C14^ix^	3.496 (3)	H14A···C18^ix^	3.0700
C15···C15^ix^	3.435 (3)	H14A···C19^ix^	3.0400
C16···C9^vii^	3.465 (3)	H14A···C20^ix^	2.9700
C16···C8^vii^	3.509 (3)	H14B···C10	2.8600
C19···C14^ix^	3.568 (4)	H14B···H10	2.4000
C20···C14^ix^	3.377 (4)	H14B···H16	2.3600
C2···H18^iv^	2.8800	H16···O2^vii^	2.7200
C4···H7	2.7500	H16···C7^vii^	3.0300
C7···H4	2.7800	H16···C8^vii^	2.9100
C7···H16^v^	3.0300	H16···C9^vii^	2.7400
C8···H16^v^	2.9100	H16···H14B	2.3600
C9···H3^ii^	2.7200	H17···C10^vii^	2.9800
C9···H16^v^	2.7400	H18···O1^xii^	2.8100
C9···H01	2.4500	H18···C2^xii^	2.8800
C10···H17^v^	2.9800	H19···H6^x^	2.5000
C10···H14B	2.8600	H20···H14A	2.5900
			
C11—O3—C14	117.99 (16)	C17—C18—C19	120.2 (2)
C2—O1—H3	109.00	C18—C19—C20	119.9 (2)
C3—N1—C7	128.73 (17)	C15—C20—C19	120.79 (18)
C7—N1—H01	112.00	C2—C1—H1	120.00
C3—N1—H01	119.00	C6—C1—H1	120.00
C2—C1—C6	120.0 (2)	C3—C4—H4	120.00
C1—C2—C3	119.26 (18)	C5—C4—H4	120.00
O1—C2—C3	115.96 (15)	C4—C5—H5	120.00
O1—C2—C1	124.8 (2)	C6—C5—H5	120.00
C2—C3—C4	120.16 (16)	C1—C6—H6	120.00
N1—C3—C4	124.50 (18)	C5—C6—H6	120.00
N1—C3—C2	115.34 (16)	N1—C7—H7	118.00
C3—C4—C5	120.0 (2)	C8—C7—H7	118.00
C4—C5—C6	120.0 (2)	C9—C10—H10	120.00
C1—C6—C5	120.60 (17)	C11—C10—H10	120.00
N1—C7—C8	123.86 (18)	C11—C12—H12	120.00
C9—C8—C13	118.82 (15)	C13—C12—H12	120.00
C7—C8—C9	121.60 (18)	C8—C13—H13	119.00
C7—C8—C13	119.54 (18)	C12—C13—H13	119.00
O2—C9—C8	119.83 (14)	O3—C14—H14A	110.00
O2—C9—C10	122.04 (17)	O3—C14—H14B	110.00
C8—C9—C10	118.12 (18)	C15—C14—H14A	110.00
C9—C10—C11	120.61 (18)	C15—C14—H14B	110.00
C10—C11—C12	121.45 (16)	H14A—C14—H14B	108.00
O3—C11—C12	113.76 (18)	C15—C16—H16	119.00
O3—C11—C10	124.78 (18)	C17—C16—H16	119.00
C11—C12—C13	119.2 (2)	C16—C17—H17	120.00
C8—C13—C12	121.81 (19)	C18—C17—H17	120.00
O3—C14—C15	107.49 (17)	C17—C18—H18	120.00
C14—C15—C16	120.87 (18)	C19—C18—H18	120.00
C14—C15—C20	121.07 (16)	C18—C19—H19	120.00
C16—C15—C20	118.05 (18)	C20—C19—H19	120.00
C15—C16—C17	121.4 (2)	C15—C20—H20	120.00
C16—C17—C18	119.7 (2)	C19—C20—H20	120.00
			
C14—O3—C11—C10	−6.0 (2)	C13—C8—C9—O2	−179.46 (15)
C14—O3—C11—C12	173.65 (15)	C13—C8—C9—C10	2.1 (2)
C11—O3—C14—C15	−169.43 (14)	C7—C8—C13—C12	175.87 (16)
C7—N1—C3—C2	−179.48 (16)	C9—C8—C13—C12	−1.5 (2)
C7—N1—C3—C4	0.4 (3)	O2—C9—C10—C11	−179.13 (15)
C3—N1—C7—C8	178.54 (16)	C8—C9—C10—C11	−0.7 (2)
C6—C1—C2—O1	179.47 (17)	C9—C10—C11—O3	178.27 (15)
C6—C1—C2—C3	−0.8 (3)	C9—C10—C11—C12	−1.3 (3)
C2—C1—C6—C5	−0.9 (3)	O3—C11—C12—C13	−177.70 (15)
O1—C2—C3—N1	1.9 (2)	C10—C11—C12—C13	1.9 (3)
O1—C2—C3—C4	−177.97 (15)	C11—C12—C13—C8	−0.5 (3)
C1—C2—C3—N1	−177.80 (15)	O3—C14—C15—C16	−107.2 (2)
C1—C2—C3—C4	2.3 (2)	O3—C14—C15—C20	73.9 (2)
N1—C3—C4—C5	178.08 (15)	C14—C15—C16—C17	−180.0 (2)
C2—C3—C4—C5	−2.0 (2)	C20—C15—C16—C17	−1.1 (3)
C3—C4—C5—C6	0.3 (3)	C14—C15—C20—C19	179.4 (2)
C4—C5—C6—C1	1.2 (3)	C16—C15—C20—C19	0.4 (3)
N1—C7—C8—C9	−0.1 (3)	C15—C16—C17—C18	0.6 (4)
N1—C7—C8—C13	−177.38 (16)	C16—C17—C18—C19	0.4 (4)
C7—C8—C9—O2	3.2 (2)	C17—C18—C19—C20	−1.0 (4)
C7—C8—C9—C10	−175.25 (16)	C18—C19—C20—C15	0.6 (4)

Symmetry codes: (i) −*x*, *y*−1/2, −*z*+1/2; (ii) −*x*, *y*+1/2, −*z*+1/2; (iii) −*x*+1, *y*+1/2, −*z*+1/2; (iv) *x*, −*y*−1/2, *z*−1/2; (v) *x*, *y*−1, *z*; (vi) −*x*+1, −*y*−1, −*z*+1; (vii) *x*, *y*+1, *z*; (viii) −*x*+1, *y*−1/2, −*z*+1/2; (ix) −*x*, −*y*+1, −*z*; (x) *x*, −*y*−3/2, *z*−3/2; (xi) *x*, −*y*−3/2, *z*−1/2; (xii) *x*, −*y*−1/2, *z*−3/2.

### Hydrogen-bond geometry (Å, º)

Cg1 is the centroid of the C15–C20 ring.

**Table:**

*D*—H···*A*	*D*—H	H···*A*	*D*···*A*	*D*—H···*A*
N1—H01···O2	0.94	1.81	2.594 (2)	139
O1—H3···O2^i^	0.82	1.75	2.563 (3)	170
C13—H13···O3^viii^	0.93	2.53	3.309 (3)	141
C14—H14*A*···*Cg*1^ix^	0.97	2.66	3.406 (3)	134

Symmetry codes: (i) −*x*, *y*−1/2, −*z*+1/2; (viii) −*x*+1, *y*−1/2, −*z*+1/2; (ix) −*x*, −*y*+1, −*z*.
