# Supplementary figures and images for: Crystal structure of (Z)-3-benz­yloxy-6-[(2-hy­droxy­anilino)methyl­idene]cyclo­hexa-2,4-dien-1-one
Source: Acta Crystallogr Sect E Struct Rep Online. 2014 Nov 26;70(Pt 12):o1292. doi: 10.1107/S1600536814024568 (PMC4257387; doi:10.1107/S1600536814024568)

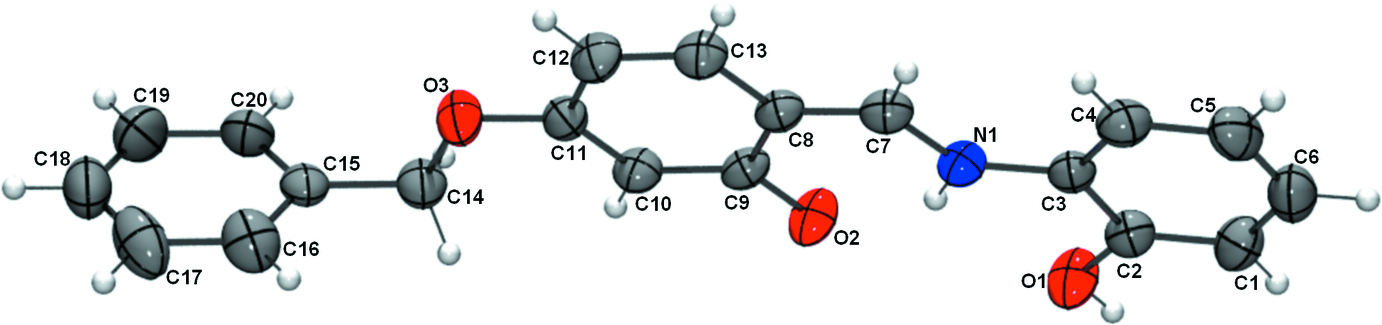

Supplement: Supplementary file 4 [file e-70-o1292-fig1.tif]

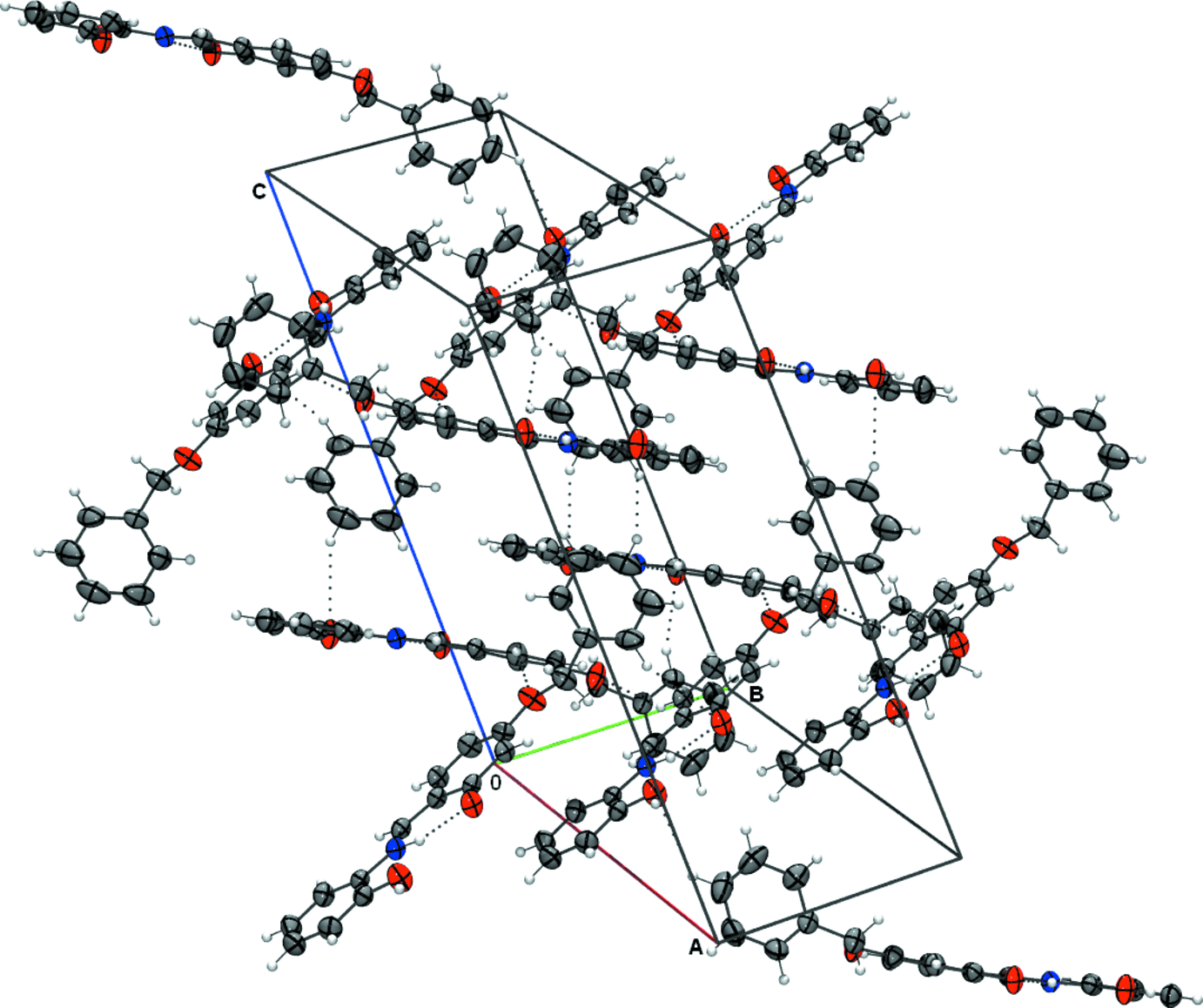

Supplement: Supplementary file 5 [file e-70-o1292-fig2.tif]
